# MicroRNA-451 Inhibits Migration of Glioblastoma while Making It More Susceptible to Conventional Therapy

**DOI:** 10.3390/ncrna5010025

**Published:** 2019-03-15

**Authors:** Daisuke Ogawa, Khairul Ansari, Michal O. Nowicki, Elżbieta Salińska, Agnieszka Bronisz, Jakub Godlewski

**Affiliations:** 1 Department of Neurosurgery, Harvey Cushing Neuro-oncology Laboratories, Brigham and Women’s Hospital, Harvard Medical School, Boston, MA 02115, USA; ohio@kms.ac.jp (D.O.); kansari@COH.org (K.A.); mnowicki@bwh.harvard.edu (M.O.N.); abronisz@bwh.harvard.edu (A.B.); 2 Department of Neurological Surgery, Kagawa University Hospital, Miki-cho, 761-0793 Kagawa, Japan; 3 Division of Neurosurgery, Beckman Research Institute, City of Hope, Duarte, CA 91010, USA; 4 Department of Neurochemistry, Mossakowski Medical Research Centre, Polish Academy of Sciences, 02-106 Warsaw, Poland; esalinska@imdik.pan.pl

**Keywords:** glioblastoma, microRNA, AMPK, invasiveness, therapy resistance

## Abstract

Malignant glioblastoma (GBM, glioma) is the most common and aggressive primary adult brain tumor. The prognosis of GBM patients remains poor, despite surgery, radiation and chemotherapy. The major obstacles for successful remedy are invasiveness and therapy resistance of GBM cells. Invasive glioma cells leave primary tumor core and infiltrate surrounding normal brain leading to inevitable recurrence, even after surgical resection, radiation and chemotherapy. Therapy resistance allowing for selection of more aggressive and resistant sub-populations including GBM stem-like cells (GSCs) upon treatment is another serious impediment to successful treatment. Through their regulation of multiple genes, microRNAs can orchestrate complex programs of gene expression and act as master regulators of cellular processes. MicroRNA-based therapeutics could thus impact broad cellular programs, leading to inhibition of invasion and sensitization to radio/chemotherapy. Our data show that miR-451 attenuates glioma cell migration in vitro and invasion in vivo. In addition, we have found that miR-451 sensitizes glioma cells to conventional chemo- and radio-therapy. Our data also show that miR-451 is regulated in vivo by AMPK pathway and that AMPK/miR-451 loop has the ability to switch between proliferative and migratory pattern of glioma cells behavior. We therefore postulate that AMPK/miR-451 negative reciprocal feedback loop allows GBM cells/GSCs to adapt to tumor “ecosystem” by metabolic and behavioral flexibility, and that disruption of such a loop reduces invasiveness and diminishes therapy resistance.

## 1. Introduction

Malignant glioblastoma (GBM), the most common primary brain tumor in adults, is among the most devastating cancers, with a median survival of approximately 15 months [1]. Current standard of care for patients includes maximal safe resection followed by irradiation and chemotherapy with temozolomide (TMZ). Invasiveness—the capability of GBM cells to infiltrate surrounding normal brain parenchyma—constitutes a major therapeutic challenge, as even extensive resection of the primary tumor mass leaves a significant number of tumor cells in the brain, leading to inevitable recurrence. The therapy resistance of GBM is greatly exacerbated by the sub-population of GBM stem-like cells (GSCs), characterized by high cellular molecular and phenotypic heterogeneity [2] which is further enhanced by microenvironmental adaptation to hypoxia and glucose deficiency [3,4]. Therefore, there is recognized need for developing novel therapy approaches that would inhibit GBM invasiveness while curbing its growth potential by overcoming therapy resistance.

GBM presents unique challenges to therapy due to its location, aggressive biological behavior, diffuse infiltrative growth and intratumoral heterogeneity [5,6,7]. GBM cells, and particularly GSCs, are characterized by chemo- and radio-therapy resistance leading to inevitable recurrence, thus targeting of GSCs offers a promising mode of GBM control. Invasion and dispersal of GBM cells into normal brain is another major source of treatment failure in humans and leads to neurologic morbidity and mortality. However, molecular and phenotypic heterogeneity of GSCs, resulting in co-existence of invasive and highly resistant sub-populations, makes single-target therapies ineffective [2,8,9,10,11,12,13]. The brain is an extremely metabolically active organ that derives energy almost entirely from glucose, which requires tight control of blood glucose homeostasis [14]. In brain tumors metabolic adaptations are critical, including aerobic glycolysis leading to even higher glucose-dependence (the Warburg effect [15]) and reduction of glucose uptake specifically in GSC sub-population attenuated their tumorigenicity [4]. Therefore, targeting adaptation to microenvironmental challenges offers novel avenues for effective anti-GBM therapy. 5’ AMP-activated protein kinase (AMPK) is a major energetic biosensor and metabolic switch that controls a broad array of biosynthetic and catabolic pathways in the cell [16]. As in certain situations of energy deficits AMPK may halt cell growth, it was first described as bona fide tumor suppressor. However, recently, a number of studies have emerged showing that AMPK enables cancer cell survival capabilities under stress [17,18,19,20,21]. Specifically, AMPK activated in solid tumors [22,23,24,25,26,27,28], was shown to promote oncogenic transformation [29,30,31,32], stemness [33,34,35], increase glucose uptake and facilitate ATP recovery [36,37,38,39,40]. AMPK is hyper-activated in GBM, AMPK inhibition resulted in decreased growth of GBM xenografts, and its activation by oncogenic events was recapitulated in rodent models of GBM [23,26,41,42,43,44]. AMPK thus provides a potent regulatory mechanism by which cancer cells temporarily halt growth on microenvironmental and therapy-inflicted challenges and it could become crucial in advanced/recurred tumors that experience metabolic and genotoxic stress [22,45]. In the last several years, important developments in cancer biology include the discovery of deregulated microRNAs and their use for therapeutic intervention [46,47,48]. Apart from microRNAs with gain and loss of function acting as tumor suppressors [49,50,51,52,53] and oncogenes [54,55] in GBM [56,57] and other malignancies [58], we identified one microRNA (miR-451) which is not deregulated in brain tumor cells per se, but is instead finely regulated by AMPK pathway. We have established the existence of a strong, reciprocal negative feedback loop between miR-451 and AMPK activity mediated by suppression of OCT1 transcription factor [59,60,61,62]. MiR-451 in turn targets simultaneously several components of the AMPK pathway and such microRNA-451/AMPK loop allows GBM cells to adapt to “stress” and nutrient deficits, promoting or suppressing brain tumor cell phenotypes based on microenvironmental contexts. If the proposed studies are successful, it would provide strong rationale for the clinical translation of a novel therapeutic strategy using miR-451 that hinder GBM cells invasion while making them more susceptible to radio/chemotherapy.

Therapeutic strategies targeting GBM require multi-target molecules to overcome heterogeneous nature of the disease. The deepened understanding of molecular and cellular mechanisms of targeted factors in discovery stage can considerably shorten expensive clinical phase and increase the chance of the development of successful approach. Testing naturally expressed microRNA candidates using heterogeneous, patient-derived cells and clinically relevant model systems to combine their effect with standard therapy is innovative way to turn phenotypic data into robust therapy-relevant discoveries.

## 2. Results

### 2.1. MicroRNA-451 Is a Potent Inhibitor of GBM Cell Motility/Migration In Vitro and Invasiveness In Vivo

**Figure 1:**
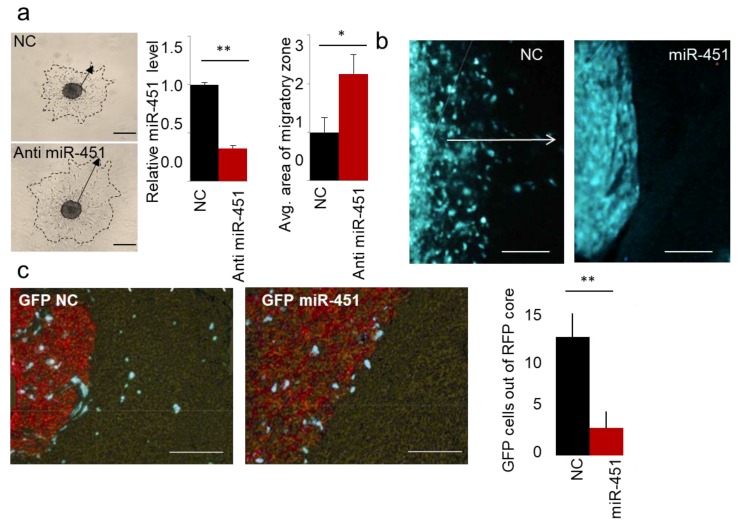
miR-451 impedes migration of GBM cells in vitro and invasiveness of GSCs in vivo. (**a**) Representative images of spheroid migration, qRT-PCR of miR-451 upon anti-miR-451 transfection, and quantification. (**b**) Representative images of intracranial xenografts of GBM12 cells expressing control GFP or GFP miR-451. White arrow depicts invasive zone. (**c**) Representative images of intracranial xenografts of GBM12 cells expressing control GFP or GFP miR-451 admixed with Red Fluorescent Protein-labeled Gli36 cells (**left**), quantification of GFP cells outside RFP core per random field (**right**). * *p*-value < 0.05, ** *p*-value < 0.01.

As we published in Reference [62], miR-451 is the most down-regulated microRNA during a 3D spheroid dispersal assay [63] in established GBM cell lines and GSCs. When miR-451 was stably overexpressed, cellular migration was markedly reduced as measured by several different assays (3D spheroid dispersal assay, Transwell and scratch/wound healing assays [62]). These results suggested that miR-451 is a functionally relevant regulator of cell migration. Conversely, when endogenous levels of miR-451 were depleted (by anti-miR-451) we observed a significant increase in cell migration (Figure 1a), demonstrating specificity of miR-451 action. As we showed, the treatment with an AMPK inhibitor (Compound C) significantly increased the expression of miR-451 and inhibited migration of GBM spheroids [61]. These results suggested the possibility that anti-migratory effect of miR-451 was in fact AMPK-dependent. Until recently it was not clear whether the observed phenomenon was relevant to in vivo phenotype. We thus tested whether miR-451 alters GSCs invasion in vivo. Firstly, we engineered invasive GSCs by overexpressing miR-451, utilizing a lentiviral expression system. We have used invasive Green Fluorescent Protein-positive GSC—GBM12 overexpressing miR-451. These cells overexpress miR-451 ~15-fold, which is similar to some non-malignant brain cells (neuroglia) and some of other GBM cells and non-malignant cells (Figure S1) and when tested by in vitro assay displayed strongly diminished migration [62]. We injected these cells intracranially into mice lacking thymic gland (athymic mice), and then sacrificed animals after three weeks. Brains were analyzed to show the distribution of GFP-positive cells.

Control GBM12 (GFP) did not form solid tumors, but rather a diffuse distribution of single cells within the brain. However, GBM12 (GFP-miR-451) cells formed well-circumscribed nodular tumors (Figure 1b). Although compelling, this finding was difficult to quantify. In the follow-up experiment we thus co-injected into the brain of athymic mice the same highly invasive but modestly proliferative GBM12 cells, either stably overexpressing GFP-miR-451 or a control vector, together with rapidly proliferating, but not infiltrative Gli36 (RFP-positive) GBM cells. As evident from Figure 1c, miR-451-expressing cells did not migrate away from the core of established tumor—in sharp contrast to control cells that appeared to be highly invasive and migrating away found from the tumor core. These results thus showed that miR-451 reduces invasion in vivo. Recently, anti-invasive effect of miR-451 has also been shown in melanoma [64], nasopharyngeal carcinoma [65], lung [66,67] and bladder [68,69] cancers.

**Figure 2:**
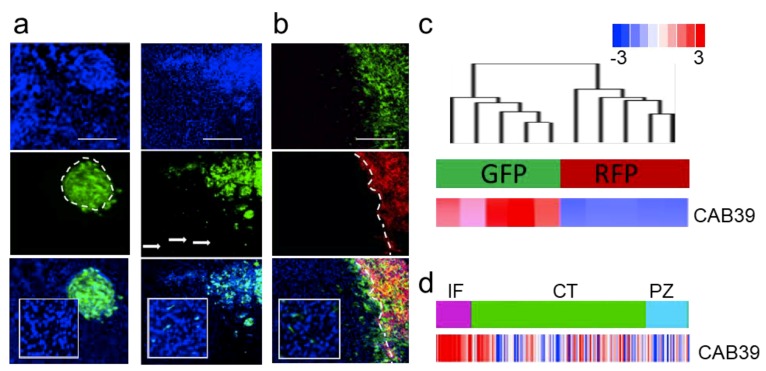
Characterization of primary GSC phenotype. (**a**) Representative images of intracranial xenografts (nodular GBM9—left, invasive GBM12—right, cells are GFP-labeled, blue—DAPI staining). (**b**) Invasive (GBM12 GFP-labeled) and nodular (GBM9 RFP-labeled) GSCs retain their phenotype in co-injection model in vivo (blue—DAPI staining). (**c**) Hierarchical clustering of gene expression in cells isolated from heterogeneous tumors (*n* = 5 independent tumor separated for GFP and RFP cells respectively) in unsupervised analysis (top cluster) and CAB39 expression (bottom bar). (**d**) CAB39 expression was retrieved from Ivy GAP database-based expression signature in different anatomic areas of GBM (IT, infiltrating tumor; CT, cellular tumor; PZ, perinecrotic zone).

We tested an extended panel of GSCs for their invasive potential. From an extensive collection of over 40 GSCs validated for their ability to invade in the brain, we estimate that 20–30% of them are capable of invading in vivo, while others form nodular tumors—both scenarios are shown on Figure 2a. Interestingly, we demonstrated that both invasive and nodular GSCs retained their characteristics both as mono- and co-cultures in vitro and when co-injected in vivo (Figure 2b, [2]). Upon co-injection these cells were explanted and sorted based on fluorescent markers. Transcriptomic analysis of molecular and cellular function of genes deregulated in these two subpopulations showed proliferative and migratory modes of the transcriptome (Figure S2a,b [70]). The analysis also demonstrated sharp difference in the transcriptomes between these two types of cells. Moreover, invasive GSCs were characterized by high expression of miR-451 target—CAB39 (Figure 2c). Finally, the expression of CAB39 was identified as anatomic site-specific signature of “infiltrating tumor”, (Figure 2d). We believe that our in vivo model is highly relevant as it recapitulates clinical characteristics of GBM: highly proliferative tumor core co-existing in the same patient with highly invasive sub-population of cells of different transcriptomic subtype [71]; and as such being extremely valuable tool to explore GBM biology with significant clinical implications.

Our data on miR-451-mediated suppression of migratory behavior of GBM cells was recently supported by findings pertaining other cancer model as well as GBM. MiR-451 inhibited the migration and invasion in vitro, as well as in vivo metastasis of hepatocellular carcinoma cells through regulating epithelial-mesenchymal transition process [72]. Importantly, Alural and colleagues demonstrated that suppression of basal levels of miR-451 in GBM cells led to increased cell migration and invasion [73]. These results underscore the relevance of miR-451 overexpression strategy as strong anti-invasive tool that do not alter significantly other phenotypic readouts of GBM cells.

### 2.2. MicroRNA-451 Sensitizes GBM Cells to Conventional Therapy

**Figure 3:**
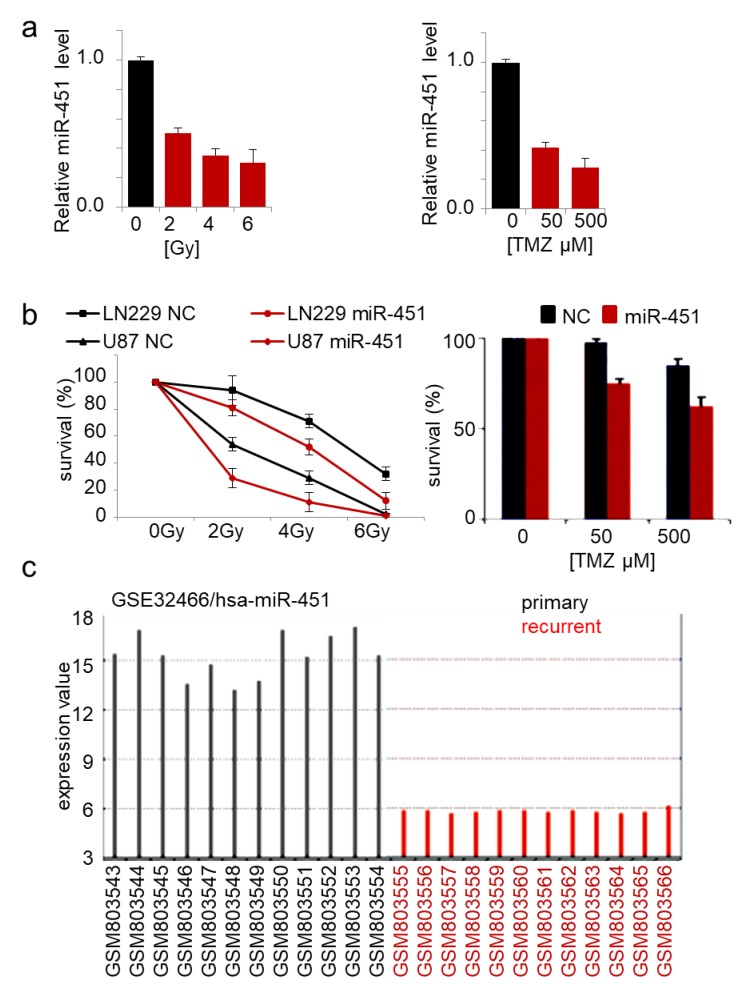
Forced expression of miR-451 sensitizes GBM cells to therapy. (**a**) miR-451 is down-regulated in cells exposed to radiation (**left**) and TMZ treatment (**right**) in GBM cells; qRT-PCR of miR-451. (**b**) miR-451 decreases survival of cells irradiated (**left**) or treated with TMZ (**right**). (**c**) miR-451 is significantly down-regulated in recurrent GBM (source: GEO accession—GSE32466).

The role of miR-451 in drug resistance of cancer cells has been reported in several malignancies. Expression of miR-451 in doxorubicin-resistant breast cancer cells increased their sensitivity to the drug [74]. Imatinib and miR-451 alone had no significant effect on GBM neurosphere formation, but in combination, led to its marked inhibition [75]. Erythropoietin-induced suppression of miR-451 in GBM led to increased cisplatin chemoresistance [73]. Overexpression of miR-451 sensitized lung cancer cells to cisplatin [76,77,78] and irradiation [79], breast cancer cells to tamoxifen and paclitaxel [80,81], and colorectal cancer cells to irinotecan [82]. We showed that GBM cells responded to TMZ treatment and irradiation by significant reduction of endogenous miR-451 expression by ~3-fold (Figure 3a), while stable overexpression of miR-451 led to significant sensitization to both therapeutic regimens (Figure 3b). Interestingly, when we queried the GEO database for the expression of microRNAs in primary vs recurrent GBM samples, miR-451 was the most significantly down regulated microRNA in recurrent GBMs (out of 251 detected microRNAs) (Figure 3c). This result underscores the importance of miR-451 downregulation in GBM cells upon treatment in order to acquire the resistance, thus allowing the recurrence. As it was demonstrated that radio- and chemo-therapy may in fact increase GBM invasiveness [83,84], we believe that miR-451 restoration concurrently with irradiation/TMZ leading to anti-migratory and pro-sensitization effect, may be a particularly relevant approach.

### 2.3. MiR-451 and Its Effector Network Are Linked to Cellular Response to Stress via AMPK Signaling to Drive the Microenvironmental Adaptation of GBM Cells/GSCs

**Figure 4:**
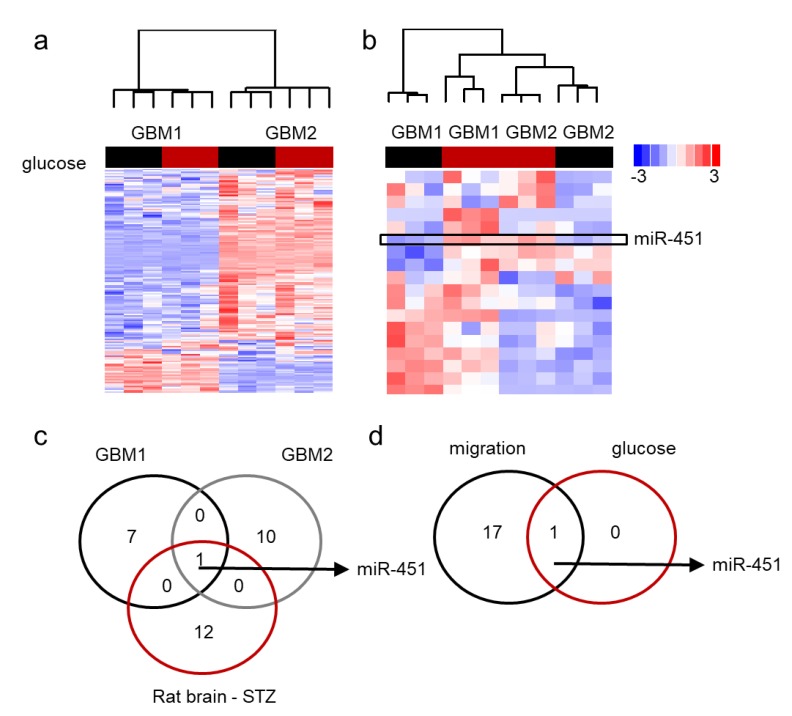
miR-451 is specifically down-regulated in low glucose in GBM cells. (**a**,**b**) Unsupervised clustering of microRNA by Nanostring analysis; red bar: high glucose, black bar: low glucose. (**a**) microRNAs significantly different in two GBM cell lines cultured for 24 h in high (+) and low (−) glucose conditions. (**b**) microRNAs significantly different in both glucose regimens in at least one cell line; box: miR-451. (**c**) Venn diagram showing number of microRNAs significantly deregulated in glucose-deprived GBM cells and brains of STZ-treated rats. (**d**) Venn diagram showing number of microRNAs significantly deregulated in glucose-deprived GBM cells and during 3D spheroid dispersal assay (3 days).

Our data has shown that miR-451 possesses significant anti-migratory effects in GBM cells and that high levels of glucose are required to maintain its expression [60]. Additionally, forced expression of miR-451 sensitizes GBM cells to conventional radio-/chemo-therapy. On the contrary, low glucose levels lead to the suppression of miR-451 levels [60,61,62]. We first determined if glucose deprivation leads to global de-regulation of microRNA expression. Figure 4a demonstrates the pattern of microRNA expression in two GBM cell lines upon glucose withdrawal by showing those microRNAs that were either significantly different between two cell lines or significantly different between high and low glucose. There was high variability of microRNA expression between the two lines and very few glucose-dependent changes. When we analyzed whether the expression of microRNAs significantly changed in low glucose in at least one cell type, it became apparent that miR-451 was the only microRNA whose expression was glucose-dependent in both cell lines: in fact, it was significantly suppressed in low glucose (Figure 4b). This finding was further supported by an experiment in which RNA was isolated from the brains of rats treated with streptozotocin (STZ)—a drug used for the induction of diabetes [85]. MiR-451 was one of a handful of microRNAs that were higher in STZ-treated (i.e., hyperglycemic) animals and the only one whose expression overlapped that of glucose-dependent microRNAs in GBM cells (Figure 4c). These results suggest that glucose-dependent microRNA rearrangements are specific rather than global. Interestingly, miR-451 was the most significantly down regulated microRNA in migrating GBM cells (Figure 4d) [62].

**Figure 5:**
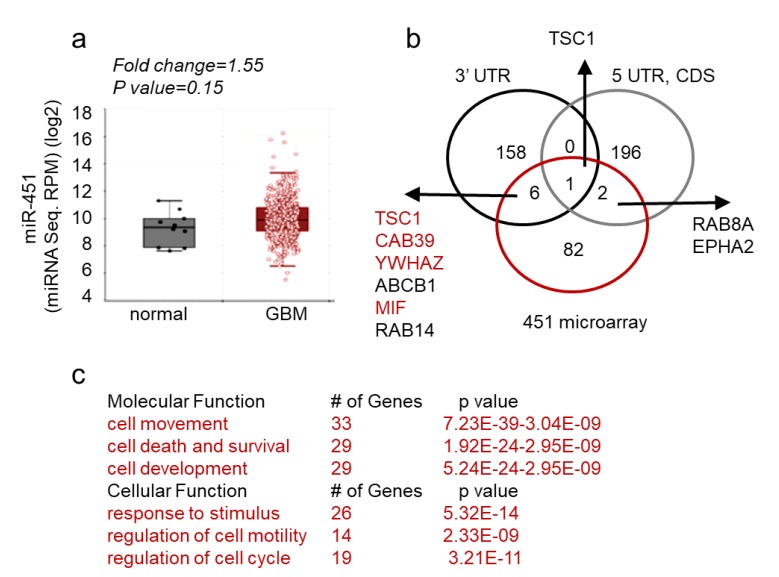
miR-451 expression in GBM and its targeted network. (**a**) MiR-451 is not significantly deregulated in GBM—microRNA sequencing of miR-451 in normal and GBM brain. Data source: TCGA. (**b**) Venn diagram showing number of genes deregulated in U87 and U251 cells stably overexpressing miR-451 (red), putative targets of miR-451 in 3′-UTRs (black) or 5′-UTRs and coding sequences (CDS) (grey)—based on TargetScan and miRWalk software. (**c**) Functional Annotation Analysis of major ontology categories of the genes down-regulated by miR-451 overexpression analyzed by IPA (Ingenuity^®^Systems) (molecular function, upper table) and GO enrichment STRING software (cellular function, bottom table).

These findings thus support a unique role of miR-451 the pathophysiology of GBM. Next, we queried both The Cancer Genome Atlas database and our collection of GBM matched samples (i.e., tumor sample with adjacent tissue devoid of gross pathology from the same individual) for the expression of miR-451. As evident from Figure 5a, miR-451 is not significantly deregulated in GBM. This finding was confirmed when we measured miR-451 levels in a collection of GSCs. It varied between Ct values 29–36, which overlapped with non-malignant neuroglia. When we stably overexpressed miR-451 in one GSC (GBM12), its levels significantly rose (from Ct 33.3 to Ct 29.8, ~11-fold) but remained within physiologically relevant range (Figure S1). To uncover the global effect of miR-451 expression, we performed a gene microarray in GBM cells stably overexpressing miR-451 and found a remarkably low number of genes (91) that were significantly down-regulated. Of these, only nine possessed a predicted miR-451 target site within their 3′-UTR, 5′-UTR or coding sequence (Figure 5b). Therefore both, microarray and in silico analysis suggest that miR-451 has an overall low number of targets and that most of deregulated transcripts are affected indirectly. Most miR-451 targets are linked to cell movement/motility, cell survival and response to endogenous stimuli (Figure 5c). The most prominent gene network among those 91 deregulated genes is the LKB1/AMPK pathway that includes targets experimentally validated by us including CAB39, YWHAZ, c-MYC [61,62] and other researchers [79,86,87,88] (Figure S3).

## 3. Discussion

The adaptation of cancer cells to the continuously changing conditions of their microenvironment as tumor progresses involves dynamic and flexible mechanisms. Tumor cells require an uninterrupted influx of nutrients and oxygen to boost growth and maintain elevated metabolism; yet, supply of blood and energy to nourish these needs is often insufficient. The adaptive mechanisms to insufficient oxygen (hypoxia) have been well described, yet the adaptations to fluctuations in glucose—the major carrier of energy are poorly understood. Elevated glucose metabolism is a hallmark of numerous solid tumors and enhanced glucose uptake was recently demonstrated to be one of the mechanisms of adaptation of glioblastoma cells to dwindled glucose supply [4]. We hypothesized that an intracellular “sensor” pathway that monitors the availability of glucose; must be overseen by the effector mechanisms that mediate adaptative response.

Cells exposed to low glucose experience shortage of ATP, increasing the [AMP]/[ATP] ratio. This event activates the 5′AMP-activated protein kinase (AMPK) complex—a highly conserved energy sensor belonging to a class of serine/threonine kinases. When cellular energy levels are decreased, AMPK is phosphorylated by LKB1 [89]. Our group has shown that miR-451, is a potent inhibitor of the AMPK signaling pathway [62] directly targeting CAB39—a necessary LKB1 co-activator. Glucose availability modulates the expression of miR-451 in glioblastoma cells. High glucose brings about high levels of miR-451, shutting-off AMPK function, inhibiting cell migration and boosting cell growth. Conversely, low glucose activates AMPK leading to diminished levels of miR-451, inhibited cell growth and enhanced migration [62]. We thus postulated the existence of an AMPK/miR-451 reciprocal negative feedback loop, mediated by glucose supply. In our recent study, we showed that the miR-451/AMPK loop is transcriptionally regulated by OCT1 transcription factor [60] whose transcriptional activity is inhibited by the phosphorylation [90]. In low glucose, activated AMPK directly phosphorylates OCT1, preventing its function and linking glucose availability to OCT1′s transcriptional function on the miR-451 promoter. Therefore, we showed that the AMPK/OCT1/miR-451/LKB1 loop provides a glucose-dependent regulatory mechanism allowing cell to adapt to fluctuating microenvironmental cues.

We demonstrated that miR-451 directly targets the 3′-UTR of CAB39 [62]—co-activator, scaffold protein for LKB1 kinase [91]. MiR-451-mediated knock-down of CAB-39 led to obliteration of the LKB1 complex and consequently reduced its activity several fold ([62]). MiR-451 acts by restraining the activity of LKB1 in a CAB-39-dependent manner and thus leads to weakened AMPK activity (diminished phosphorylation of AMPK itself, Raptor, ACC, TSC2 or increased phosphorylation of RPS6K and RPS6) in GBM cells [62]. AMPK is a cellular energy sensor conserved in all eukaryotic cells [92] and it becomes activated by stimuli that increase the cellular AMP/ATP ratio and phosphorylation by LKB1 (or less frequently by other kinases). AMPK regulates the activities of a number of key metabolic enzymes through phosphorylation. It protects cells from stresses that cause ATP depletion by switching off ATP-consuming biosynthetic pathways [93]. Overexpression of miR-451 not only reduced basal level of active AMPK, but effectively prevented proper activation of AMPK [62]. On the other hand, when we reduced the amount of glucose in the medium (15-fold from 4.5 g/L to 0.3 g/L), we observed a significant reduction in miR-451 expression in all GBM cells tested, but not in HeLa cells, [60,62] that are known for lack of functional LKB1 due to gene methylation [62,92] suggesting that intact AMPK signaling is required for repression of miR-451 in low glucose. Knock-down of AMPK α catalytic subunits in high glucose had no effect on miR-451 expression, while the same knockdown in low glucose led to de-repression of miR-451 expression [60]. Additive effect of double knock-down of α1/α2 can be explained by known functional redundancy between AMPK α subunits [93,94,95]. These results indicate a reciprocal negative feedback loop between miR-451 and AMPK, as we recently demonstrated [60]. The concept of miR-451/AMPK loop was also described in mathematical models of GBM growth/invasion [94,96,97,98,99]. We determined transcriptional mechanism that regulates miR-451—a transcription factor implicated in glucose/energy/stress signaling: OCT1, [60,90]. OCT1 has been shown to be functional in several types of cancer and to lead to increased glycolytic metabolism [90,100,101,102], (the “Warburg effect” [103]), leading cancer cells to glucose-dependency. In fact, OCT1 knock-down in GBM cells led to significant, two-fold decrease in miR-451 levels [60]. We also tested *Oct1−/−* fibroblasts that showed several-fold diminished levels of miR-451 [60]. Moreover, these cells are able to withstand medium that has no glucose—a phenotype that is also dependent on low levels of miR-451 [100]. In GBM cells with activated AMPK, OCT1 became phosphorylated and in consequence inactivated [104]. Re-introduced wild type or S335A mutant OCT1 into *Oct1−/−* fibroblasts it rescued the expression of miR-451. The reciprocal negative feedback loop between miR-451 and AMPK and the inactivation by phosphorylation of transcription factor promoting miR-451 in conditions of low glucose and AMPK activation, suggested that AMPK may be the kinase directly phosphorylating OCT1. Briefly, we demonstrated that expression of miR-451 inversely correlates with activated AMPK and that AMPK is sufficient for the phosphorylation of OCT1 at S335, thus establishing AMPK as the kinase responsible in shutting-off OCT1-mediated transcription of miR-451 in response to glucose availability [60].

Recently, it has become increasingly clear that the AMPK complex endows cancer cells with the ability to survive the exposure to stress, (including energy deficiency and genomic damage) [18], despite the fact that AMPK was historically perceived as a bona fide tumor suppressor, as it can impede cell growth. It provides a potent adaptative mechanism by which cancer cells are capable of briefly stop their growth as they face microenvironmental challenges. AMPK can be thus seen as a “contextual oncogene”, enabling cancer cells with pro-survival flexibility.

Forced expression of miR-451 under stress leads to cytotoxicity [62], as adaptative measures (energy-conserving metabolic shift and resource-seeking behavioral change) require brain tumor cell to shut down miR-451. The AMPK-dependent inactivation of transcriptional activator of miR-451 enables cancer cells with the ability to escape from metabolically stressful events/locations. MiR-451 is thus an example of a small RNA molecule that is not deregulated in brain tumor cells per se, but is instead finely regulated by promoting contrary cell phenotypes based on microenvironmental contexts. The de-regulation of miR-451/AMPK feedback loop provides a novel avenue for therapeutic intervention that would simultaneously target two major obstacles to the development of successful anti-glioblastoma modality: invasiveness and resistance to genotoxic therapy.

## 4. Materials and Methods

All standard experimental procedures (cell culture, engineering of stable cell lines, real-time PCR, transfections, cell assays, isolation of RNA, intracranial xenografts) were performed as described by us [2,60,62,105]. Streptozotocin treatment was performed as described in References [85,106].

### 4.1. Statistical Analysis

Data are expressed as mean ± SD. Statistical analyses were performed using the unpaired two-tailed Student’s *t* test from the GraphPad Prism software. Differences were considered statistically significant at *p* < 0.05. All microscopy-based assays were edited/quantified using ImageJ using the Analyze Particles function of binary images with an automatic threshold. One-way ANOVA followed by Bonferroni’s test was conducted to test for significance among multiple groups. *p* < 0.05 was considered significant. All significantly deregulated transcripts were visualized via a heatmap and analyzed using dChip software with the Statistical R package. An unpaired, two-tailed *t* test was used to compare two groups. The list of putative miR-451 target mRNAs was generated based on Target Scan v. 7.1 (http://www.targetscan.org/vert_71/).

### 4.2. Bioinformatic Analysis

Functional bioinformatics analyses were performed using Qiagen’s Ingenuity Pathway Analysis (IPA; www.qiagen.com/ingenuity). Experimental and clinical data were analyzed using the GBM-BioDP [107]. Clinical data were downloaded from the TCGA data portal (https://tcga-data-nci-nih-gov.ezp-prod1.hul.harvard.edu/). Gene expression in the various anatomical regions of glioblastoma was analyzed using the Ivy Glioblastoma Atlas Project (http://glioblastoma.alleninstitute.org/). Level 3 microRNA expression data (unc.edu_GBM.H-miRNA_8 × 15 K. Level_3.1.8.0) from 479 glioblastomas were obtained from TCGA [108].

### 4.3. Transcriptome Analysis

Whole Human Genome Oligo Microarray was performed by Arraystar as described in Reference [109]. The data were deposited into Gene Expression Omnibus with accession number GSE89501). Analysis of miR-451 in primary and recurrent samples was done based on microarray data deposition with accession number GSE32466.

### 4.4. In Vivo Experiments

Female, 6- to 8-week-old immunodeficient athymic nude mice were purchased from Envigo. For intracranial tumor injection, a total of 2.5 × 10^4^ GFP and 2.5 × 10^4^ Tomato (as previously described [70]) expressing stable cells were injected and allowed for tumor establishment. Animals were sacrificed at 6-day time point after injection to determine tumor growth using brain sections imaged by confocal microscope (Zeiss LSM710), or brain tissue was immediately processed. Briefly after explanting, fresh tumor was enzymatically dissociated using a gentle papain-based brain tumor dissociation kit (Miltenyi Biotec). Large pieces of debris were removed with a strainer, and dissociated cells were layered onto density gradient and processed accordingly to the manufacturer’s protocol. For depletion of mouse cells we labeled suspension cells with a cocktail of monoclonal antibodies conjugated with MicroBeads and then loaded onto a column placed in the magnetic field of separator and eluted of human cells that were sorted by flow cytometry (using GFP/RFP). RNA isolated were subjected for gene expression analysis using microarray as previously described [105].

## Supplementary Materials

The following are available online at https://www.mdpi.com/2311-553X/5/1/25/s1, Figure S1, Figure S2, Figure S3.
