# Supplementary material for: MicroRNA-451 Inhibits Migration of Glioblastoma while Making It More Susceptible to Conventional Therapy
Source: Noncoding RNA. 2019 Mar 15;5(1):25. doi: 10.3390/ncrna5010025 (PMC6468936; doi:10.3390/ncrna5010025)

non-coding RNA

MDPI

**Supplementary materials:**

**Supplementary Figure 1: The expression of miR-451 is variable in primary GBM cells.** NG – neuroglia; GBM 12 carrying either control (NC) or miR-451 expressing vector. Ct values of qRT-PCR are shown.

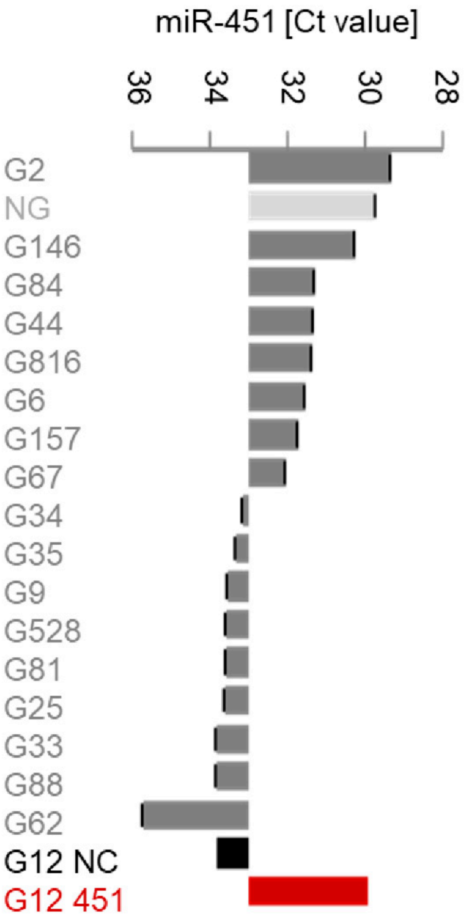

**Supplementary Figure 2.** Gene expression analysis in nodular (**A**) vs. invasive (**B**) GSCs (n=8 independent GSCs, 4 per subclass; p< 0.05, fold>2) showing their cellular localization, network and function.

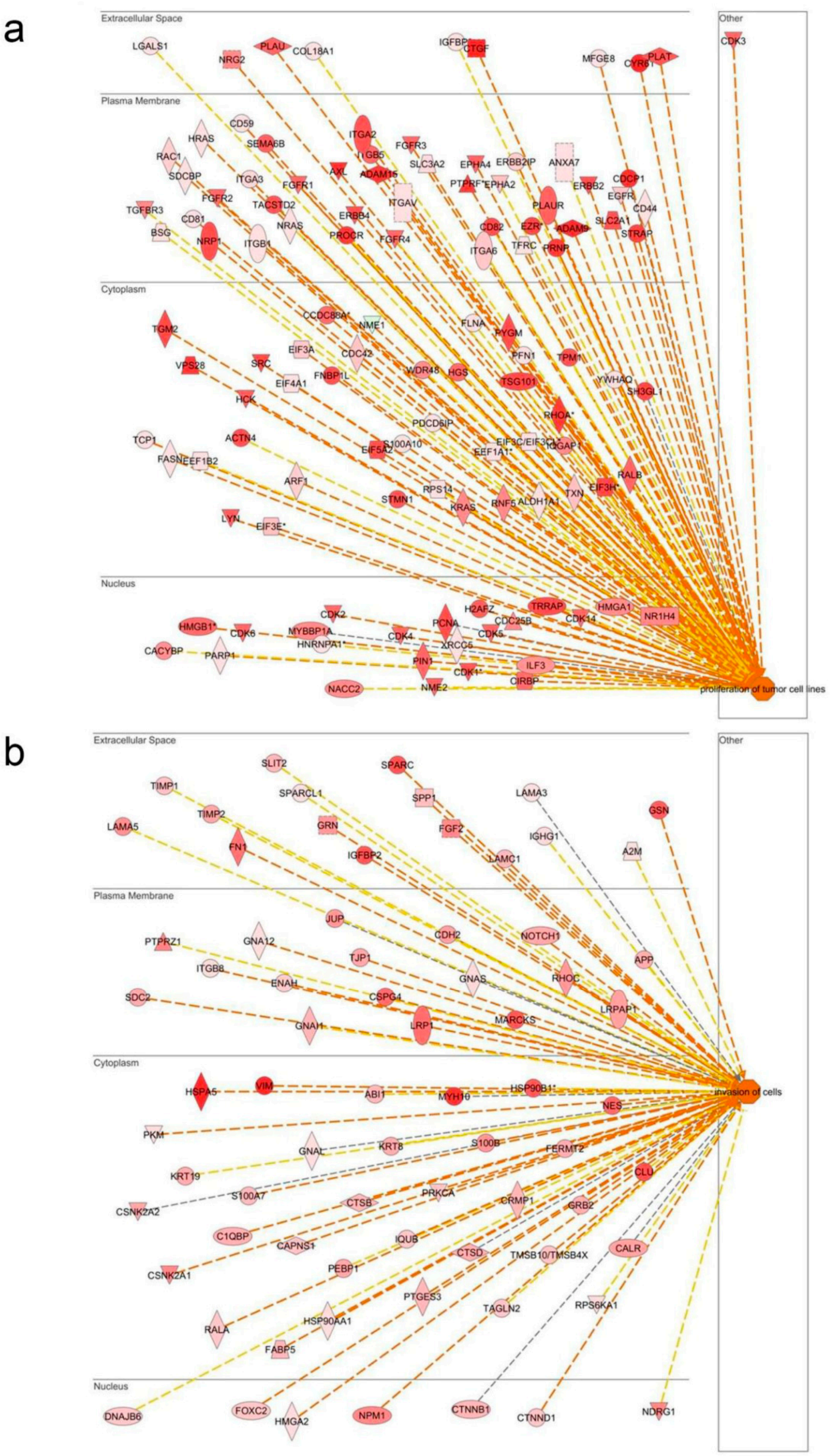

**Supplementary Figure 3.** AMPK network is the most prominent among 91 targeted genes (see Figure 5. (STRING software). Experimentally validated miR-451 targets are indicated by solid black line.

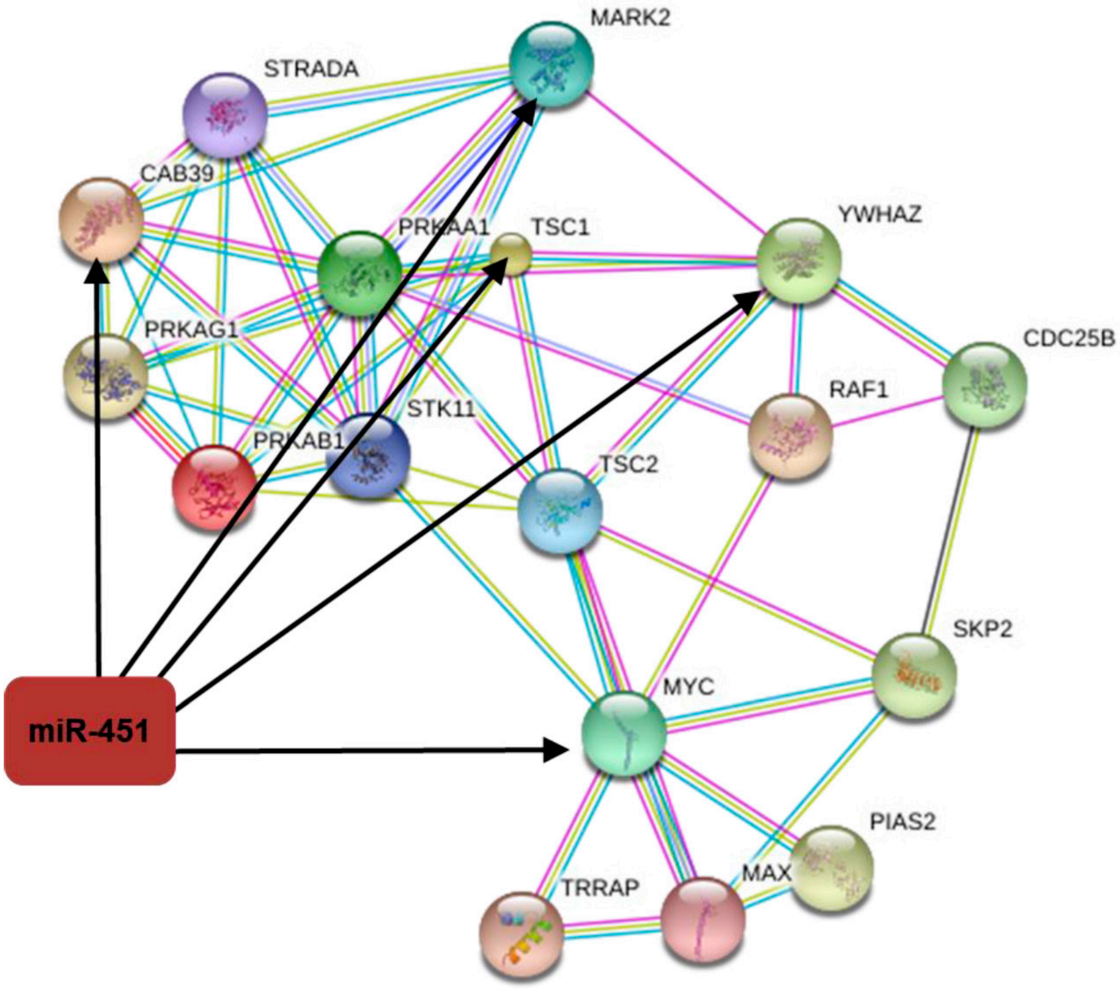

Supplement: Supplementary file 1 [file ncrna-05-00025-s001.pdf]
